# Swansea Summer School of Automatic Chemical Analysis 1981

**DOI:** 10.1155/S1463924681000333

**Published:** 1981

**Authors:** J. P. Chester


The area of computer applications is a major area of concern to management and one where close control is required. Teaching chemists to programme a computer can be a great advantage but this knowledge is best used if the chemist uses it to broaden his horizon so that he can precisely specify the requirements for the analysis or instruments to computer specialists who can then develop the software correctly and economically. A chemist may well be able to write a simple programme but tailoring the software to meet such constraints as continuous unattended operation is more difficult and best left for experts in this area.

*P.B. Stockwell*

### Swansea Summer School of Automatic Chemical Analysis 1981

With topics from operational amplifiers to continuous flow analysis and from microprocessors to automated instrumental analysis, this years Summer School at University College, Swansea gave delegates an overview of the entire field of automation in chemical and clinical laboratories. It was a large and complex programme, bringing together experts from many countries to describe their work to over fifty representatives from as many different fields. A total of eighteen lectures were scheduled for the morning sessions, with practical work and tutorials in the afternoons.

The difficult task of reconciling the requirements of such a diverse group was solved quite elegantly. A straw poll was held at the begining of the week to select tutorial topics, and six of these were then scheduled simultaneously. Despite the opportunities for confusion thus offered, the efficient organisation ensured that all delegates had access to the expertise in which they were interested.

In addition, ten instrument companies allowed participants to use their latest products in the practical sessions. When one recalls the advice repeated by many of the experienced lecturers to buy rather than build, these sessions gave invaluable hands-on experience of the available hardware.

Highlights can only be a personal choice. My choice, in no particular order, would include Prof. Gary Horlick from the University of Alberta describing the architecture of microprocessors, and Prof. D.L. Massart from Vrije Universiteit, Brussels showing how to make sense of the mass of data generated by the use of automatic analysis. With the emphasis on data collection and analysis during the week, the timely reminder from Prof. Jack Betteridge that the objective should be to do the chemistry better or in a novel way was needed. Finally, a mention for Dr. Kent Stewart who, not content with the invention of automated multiple flow injection analysis, tentatively suggested that his most recent experiments with interfacing computers with analytical instruments may be the beginning of robotics!

*Dr. J.P. Chester*

SHIMADZU

High performance
but low cost G.C. and integrators

Ideal for use in research
& quality control laboratories

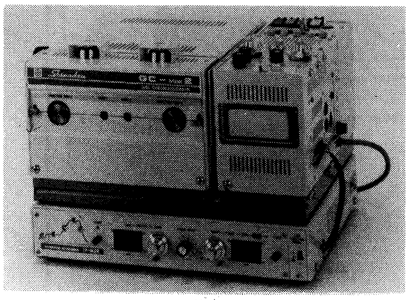

GC MINI 2

- Dual FIDs.
- Wide range amplifier.
- Independent temperature control for oven and injection/detector heaters.
- Full flow controls.

PRICE FROM £2200.
Plus VAT.

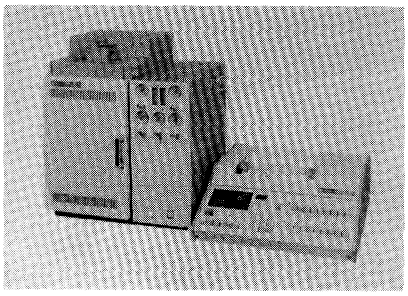

GC RIA SERIES

- Choice of detectors
- Built in computing integrator
- All electronic controls programmed from data processor
- Full flow controls and injector purging system.

PRICE FROM £5600
Plus VAT.

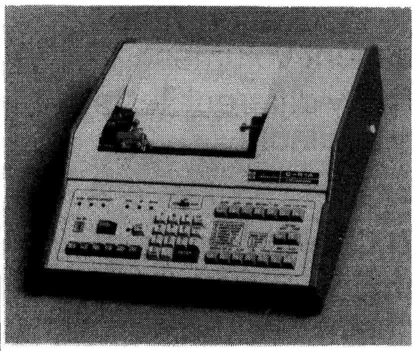

COMPUTING INTEGRATORS
CRIA

- Fully alphanumeric printer plotter.
- Performs all calculations, including 2 point calibration.
- Two month memory protection against power failure.

PRICE £2195 Plus VAT.
CEIB.
Printer version of above
PRICE £1550 Plus VAT.

*Full colour brochures available on request from:—*

DYSON INSTRUMENTS

Sunderland House, Station Road,
Hetton, Houghton Le Spring,
Tyne & Wear, DH5 0AT
Telephone: (0783) 260452, 260433

